# Maternal cold exposure induces distinct transcriptome changes in the placenta and fetal brown adipose tissue in mice

**DOI:** 10.1186/s12864-021-07825-6

**Published:** 2021-07-03

**Authors:** Sujoy Ghosh, Chul-Hong Park, Jisu Lee, Nathan Lee, Rui Zhang, Clara Huesing, Dorien Reijnders, Jennifer Sones, Heike Münzberg, Leanne Redman, Ji Suk Chang

**Affiliations:** 1 Genomics and Bioinformatics Core, Pennington Biomedical Research Center, 6400 Perkins Road, Baton Rouge, USA; 2 Centre for Computational Biology, Duke-NUS Medical School, Singapore, Singapore; 3 Gene Regulation and Metabolism, Pennington Biomedical Research Center, 6400 Perkins Road, Baton Rouge, Louisiana, 70808 USA; 4 Leptin Signaling in The Brain, Pennington Biomedical Research Center, Baton Rouge, Louisiana, USA; 5 Louisiana State University School of Veterinary Medicine, Baton Rouge, Louisiana, USA; 6 Reproductive Endocrinology and Women’s Health, Pennington Biomedical Research Center, Baton Rouge, Louisiana, USA

**Keywords:** Brown adipose tissue thermogenesis, Fetal brown adipogenesis, Placenta, RNA-sequencing, Gene expression, Maternal-fetal crosstalk

## Abstract

**Background:**

Brown adipose tissue (BAT) is specialized to dissipate energy in the form of heat. BAT-mediated heat production in rodents and humans is critical for effective temperature adaptation of newborns to the extrauterine environment immediately after birth. However, very little is known about whether and how fetal BAT development is modulated in-utero in response to changes in maternal thermal environment during pregnancy. Using BL6 mice, we evaluated the impact of different maternal environmental temperatures (28 °C and 18 °C) on the transcriptome of the placenta and fetal BAT to test if maternal cold exposure influences fetal BAT development via placental remodeling.

**Results:**

Maternal weight gain during pregnancy, the average number of fetuses per pregnancy, and placental weight did not differ between the groups at 28 °C and 18 °C. However, the average fetal weight at E18.5 was 6% lower in the 18 °C-group compared to the 28 °C-group. In fetal BATs, cold exposure during pregnancy induced increased expression of genes involved in de novo lipogenesis and lipid metabolism while decreasing the expression of genes associated with muscle cell differentiation, thus suggesting that maternal cold exposure may promote fetal brown adipogenesis by suppressing the myogenic lineage in bidirectional progenitors. In placental tissues, maternal cold exposure was associated with upregulation of genes involved in complement activation and downregulation of genes related to muscle contraction and actin-myosin filament sliding. These changes may coordinate placental adaptation to maternal cold exposure, potentially by protecting against cold stress-induced inflammatory damage and modulating the vascular and extravascular contractile system in the placenta.

**Conclusions:**

These findings provide evidence that environmental cold temperature sensed by the mother can modulate the transcriptome of placental and fetal BAT tissues. The ramifications of the observed gene expression changes warrant future investigation.

**Supplementary Information:**

The online version contains supplementary material available at 10.1186/s12864-021-07825-6.

## Background

While white adipose tissue (WAT) stores energy as fat, brown adipose tissue (BAT) is specialized for dissipating energy as heat. BAT thermogenesis is achieved by mitochondrial uncoupling protein 1 (UCP1) that dissipates the proton gradient generated by the electron transport chain in the form of heat [1, 2]. In rodents and small mammals, including infant humans, BAT-mediated heat production enables newborns to adapt to the extrauterine environment after birth. Recent studies in rodents and humans further indicate that BAT also plays a critical role in regulating metabolic health later in life.

Lineage-tracing studies have revealed that progenitors in the central dermomyotome [3] and Pax7-negative progenitors in the epaxial dermomyotome [4] give rise to the brown adipocytes in the interscapular BAT during early stages of embryogenesis. BAT is first detectable at embryonic day 15.5 and then increases in size until birth [5, 6]. During this stage of development, BAT is derived from rapidly proliferating progenitors that express myogenic markers such as Myf5, MyoD, and Myogenin [7, 8]. After E16.5, myogenic gene expression declines as the progenitors differentiate into mature brown adipocytes. Transcriptional factor PRDM16 is a key regulator that promotes the brown adipocyte lineage from a bidirectional progenitor [9–11].

In rodents, maternal body temperature is maintained constant by BAT thermogenesis that is tightly controlled via the sympathetic nervous system in response to changes in environmental temperature. The decreased demand for thermogenesis at warm environments results in the entry of BAT into an inactive or dormant state [2], whereas the increased need for thermogenesis at cold environments enhances the thermogenic capacity of BAT. Fetal thermoregulation in the uterus is maternally dependent [12]. The maternal body provides heat to the fetus via the placenta and the umbilical circulation. However, immediately after birth, the neonate must rapidly increase BAT-mediated heat production, which is coupled to lipolysis and fatty acid oxidation in BAT [2], to adapt to the extrauterine environment that is often colder than the intrauterine environment. However, whether maternal thermal environments influence fetal BAT development is largely unknown. Here we hypothesized that maternal adaption to environmental temperature also influences fetal BAT development for ensuring offspring better prepared to the environment experienced by the mother. The placenta is the functional interface between the mother and the fetus and plays a key role in fetal development. Thus, we further hypothesized that maternal thermal environments could influence fetal BAT development via placental remodeling.

To test our hypotheses, we housed pregnant female mice to near thermoneutral (28 °C) or mild cold (18 °C) temperature during pregnancy and evaluated the impact of different maternal environmental temperatures on the transcriptome of the placenta and fetal BAT. Our results provide insights into the molecular mechanisms by which maternal thermal environments influence placental function and fetal BAT development.

## Results

### Effects of maternal cold exposure on placental and fetal weight

**Fig. 1:**
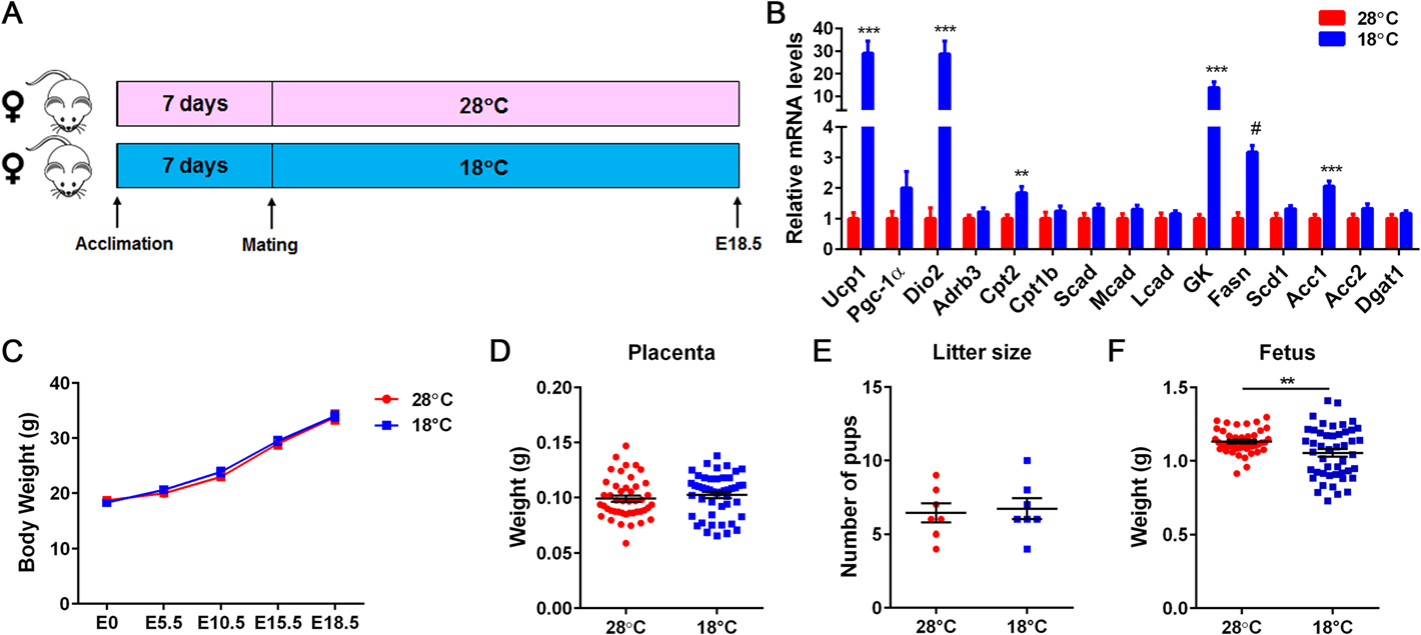
Effects of maternal cold exposure on placental and fetal weight. (**A**) Scheme for maternal exposure to two different environmental temperatures. Female C57BL/6 J mice were acclimated at 28 °C (*n* = 7) or 18 °C (*n* = 7) for 1 week and mated with males. Pregnant females were maintained at respective temperature until embryonic day 18.5. Animal images are taken from freely available online pictures in the public domain. (**B**) Gene expression analysis of BAT samples from dams exposed at 28 °C (*n* = 7) or 18 °C (*n* = 7). (**C**) Body weight of pregnant dams during pregnancy (*n* = 7 per group). (**D**) Weight of placentas (7 litters per group). (**E**) Litter size per pregnancy. (**F**) Weight of fetuses (7 litters per group). All data are presented as the mean ± SEM. **P < 0.01, ***P < 0.001, ^#^*P* < 0.0001 determined by Student’s *t* test

In mice, the thermoneutral zone lies at approximately 30 °C at which BAT thermogenesis is lowest, whereas the mice housed at room temperature (22–23 °C) experience mild cold stress and must increase their metabolism and BAT thermogenesis to defend their body temperature [13, 14]. To evaluate the effects of maternal thermal environments on the placenta and fetal BAT development, female mice were acclimated for 1 week at near thermoneutrality (28 °C) or 18 °C prior to mating with male mice, and pregnant females were maintained at their respective temperature until embryonic day 18.5 (Fig. 1A). Pregnant dams housed at 28 °C or 18 °C exhibited no difference in weight gain throughout the pregnancy (Fig. 1C). Maternal cold exposure significantly elevated BAT thermogenesis in 18 °C-exposed dams compared to 28 °C-exposed dams, as evidenced by upregulation of key genes involved in thermogenesis, fatty acid transport, and lipogenesis (Fig. 1B). Maternal cold exposure did not alter placental weight (Fig. 1D) and the average number of fetuses per pregnancy (Fig. 1E); however, the average fetal weight at embryonic day18.5 was 6% lower in the 18 °C-group compared to the 28 °C-group (Fig. 1F).

### Sequencing profile of fetal BAT and placental transcriptome

**Fig. 2:**
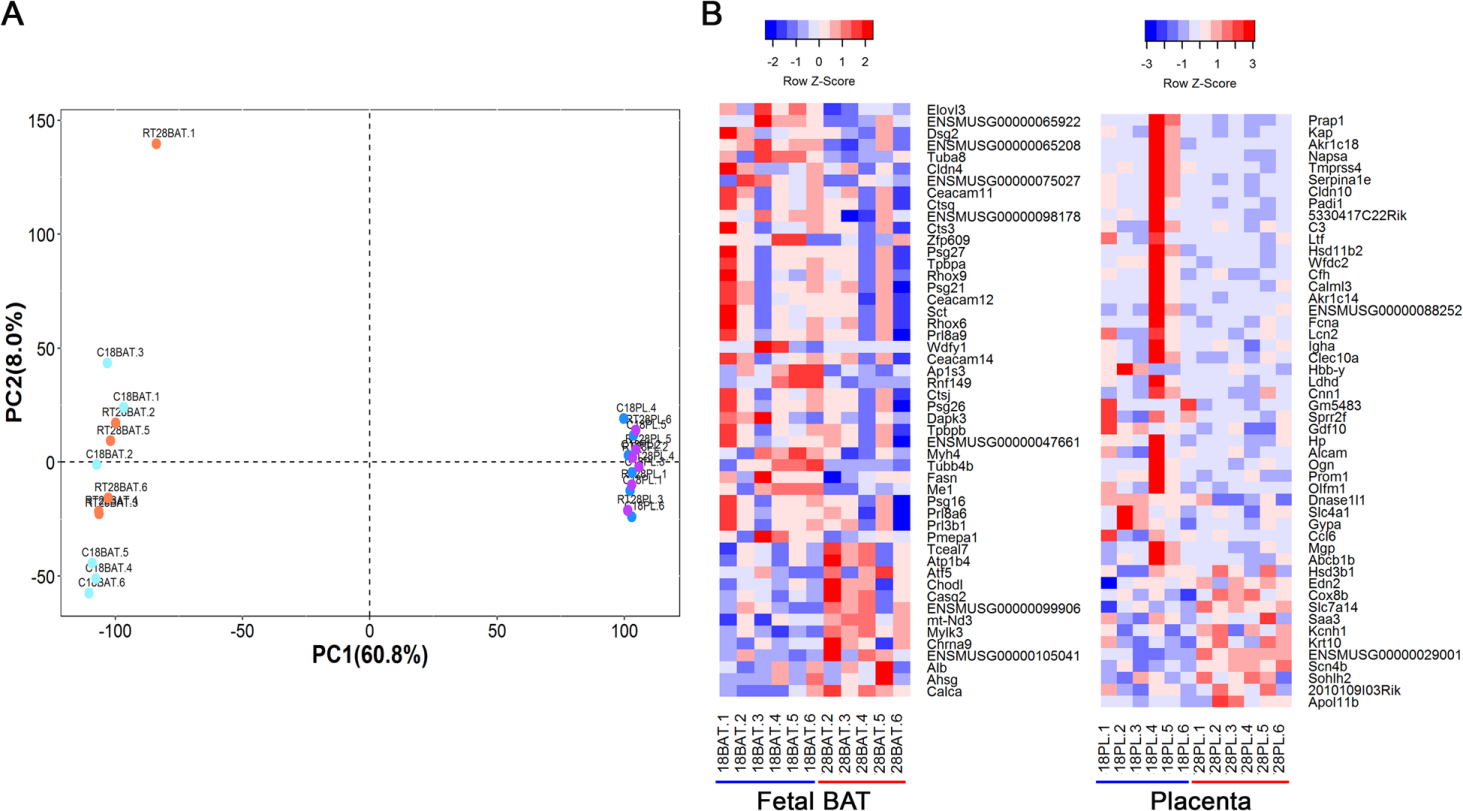
Transcriptome analysis of placental and fetal BAT tissues. (**A**) Principal components analysis (PCA) of gene expression data. 28 °C-fetal BAT (orange), 18 °C-fetal BAT (cyan), 28 °C-placenta (purple), and 18 °C-placenta (blue). (**B**) Heat maps of fetal BAT and placental transcriptome that is differentially regulated by maternal thermal environments. The expression profiles of the top 50 genes are sorted by FDR, using a cut-off FDR < 0.05. Upregulated genes are shown in red and downregulated genes are shown in blue, with gradient colors representing the level of expression (deeper shades for higher degree of differential expression)

To assess the effects of two different maternal thermal environments on the transcriptome of placental and fetal BAT tissues, placental (6 biological replicates per temperature) and fetal BAT tissues (6 pooled biological replicates per temperature) were collected at E18.5 from dams exposed to 28 °C or 18 °C for the transcriptome profiling of all mRNAs via high-throughput sequencing. Sequence reads were aligned to the GRCm38 mouse reference genome. In total, 13,068 and 13,615 mRNAs were obtained from fetal BAT and placental tissues, respectively. To analyze the distribution of gene expression, principal components analysis (PCA) was performed on the gene expression datasets of placental and fetal BAT tissues. The PCA plot resolved only into two groups primarily by the tissue of origin (Fig. 2A). One sample (fetal BAT_28°C.1) behaved as an outlier and was excluded from further analysis. Differential gene expression analysis between the 18 °C and 28 °C samples identified 67 genes in fetal BAT and 95 genes in placenta at FDR < 0.05, respectively (Supplementary Table 1). The expression profiles of the top 50 genes (sorted by FDR) for fetal BAT and placenta was visualized through clustered heatmaps (Fig. 2B).

### Differential gene expression analysis of fetal BAT

**Fig. 3:**
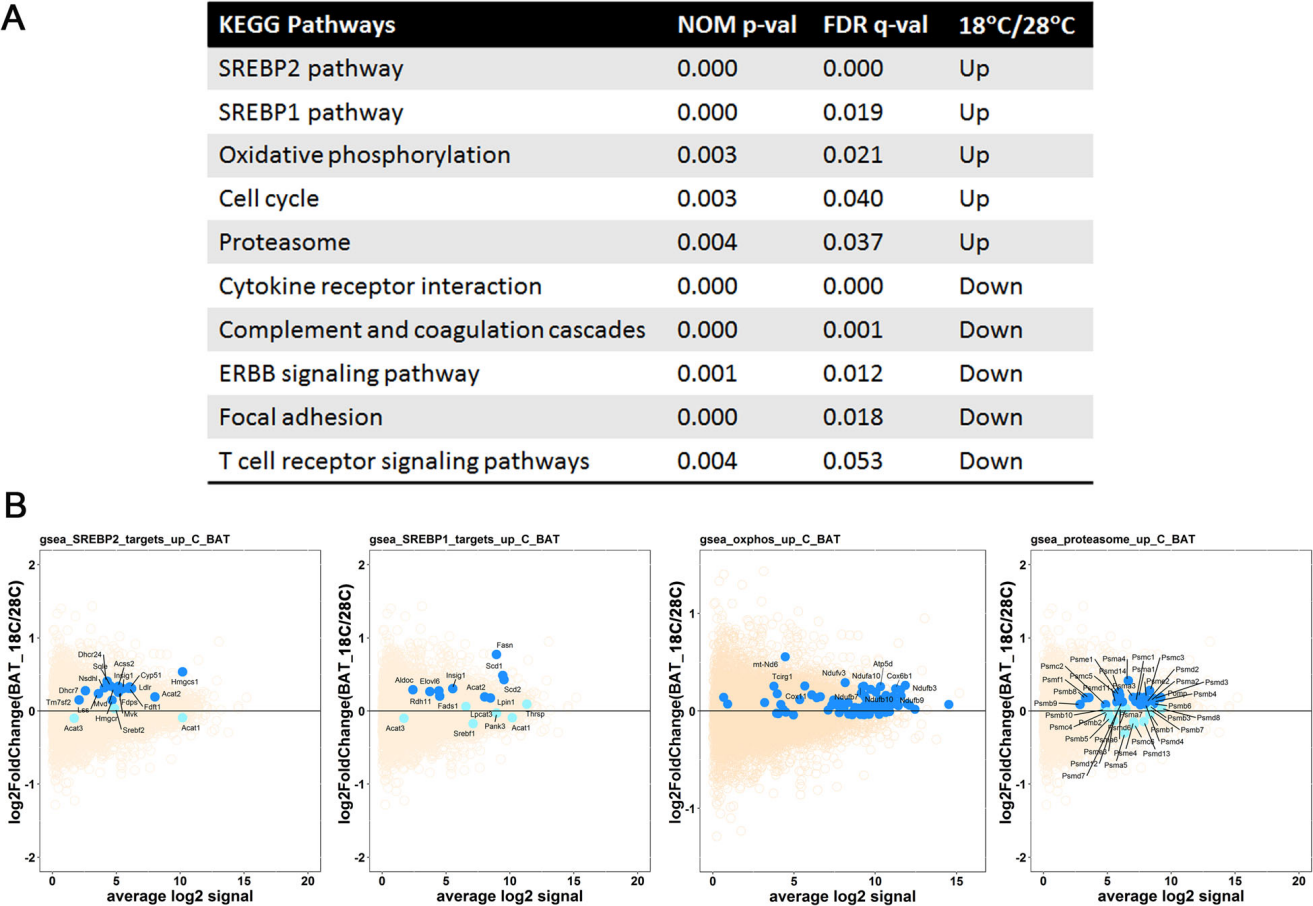
Effect of maternal cold exposure on fetal BAT gene expression. (**A**) Gene set enrichment analysis (GSEA) showing the pathways enriched in upregulated and downregulated genes in fetal BAT in a cold-dependent manner. (**B**) Mean-average (MA) plot analysis. Pathway genes contributing to pathway enrichment are shown in blue, pathway genes not contributing to pathway enrichment are shown in cyan, and non-pathway genes are shown in tan

In order to identify metabolic pathways that may be differentially affected by maternal cold in fetal BAT samples, we conducted gene-set enrichment analysis (GSEA) of the gene expression data. A total of 6 KEGG pathways were upregulated and 15 pathways downregulated in 18 °C fetal BAT samples at a pathway enrichment FDR < 0.1 (Supplementary Table 2). Top 5 upregulated and top 5 downregulated pathways for fetal BAT samples are listed in Fig. 3A and represent diverse biological functions includi*ng lipid metabolism (SREBP1/SREBP2 pathways), oxidative phosphorylation, proteasome, ERBB signaling, and focal adhesion,* etc. We further examined the expression patterns of the gene-members in 4 of the cold-upregulated pathways via MA plots as shown in Fig. 3B. Genes contributing to core enrichment of each pathway (ascertained through GSEA, shown in blue in the plots) were predominantly found to be upregulated at 18 °C compared to 28 °C (y-axis value > 0) compared to non-contributing pathway genes (cyan) or non-pathway genes (tan), suggesting that coordinated small changes from several pathway genes are responsible for the observed significant enrichment of the pathways. Intriguingly, the metabolic pathways associated with upregulated genes in maternal cold-exposed fetal BAT are in line with previous findings. Cold has been shown to stimulate lipid metabolism, oxidative phosphorylation, and proteasome pathways in BAT [2, 15, 16]. SREBP1/2 are transcription factors that regulate lipid biosynthesis by controlling the expression of key enzymes required for cholesterol, fatty acid, and triacylglycerol synthesis [17] and are also involved in adipocyte differentiation [18, 19]. During fetal BAT development, myogenic gene expression declines as the progenitors differentiate into mature brown adipocytes [7, 8]. Among the pathways associated with downregulated genes, the ErbB signaling pathway has been implicated in myogenic differentiation [20, 21].

**Fig. 4:**
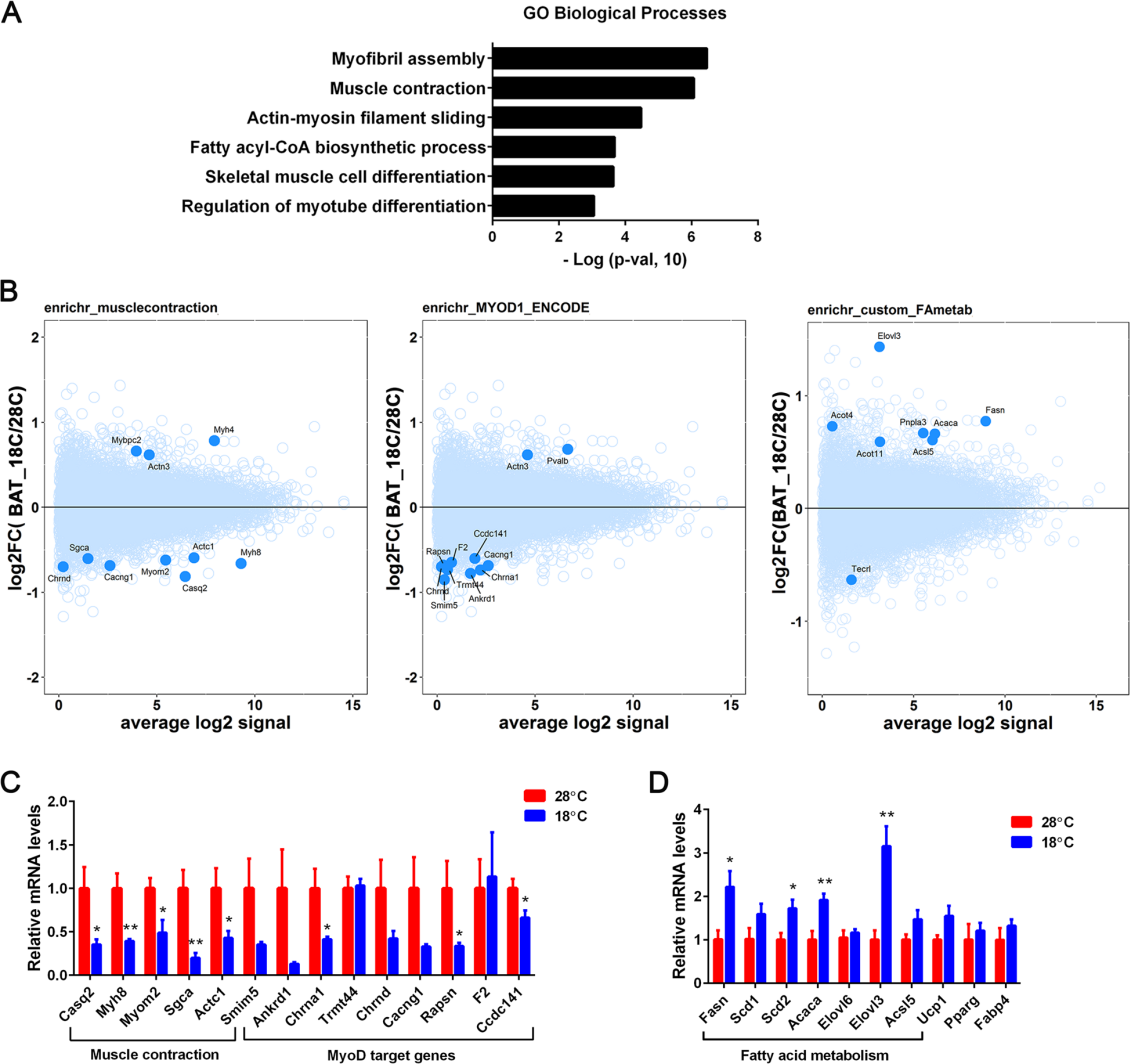
Pathways enriched in differentially regulated genes in maternally cold-exposed fetal BAT. (**A**) Gene ontology (GO) analysis. 230 differentially regulated fetal BAT genes with a nominal *p*-value ≤0.01 and absolute fold-change ≥1.5 (18 °C vs 28 °C) were used as input for Enrichr. (**B**) MA plot analysis. Pathway genes are shown in blue and non-pathway genes shown in cyan. (**C**, **D**) Quantitative real-time PCR analysis of 28 °C- and 18 °C-fetal BAT samples (*n* = 6 per group). Data are presented as the mean ± SEM. *P < 0.05, **P < 0.01 determined by Student’s *t* test

As a complementary approach to GSEA, we performed pathway over-representation analysis to examine the enrichment of pathways among 230 differentially expressed genes (nominal *p*-value ≤0.01 and absolute fold-change ≥1.5) in fetal BAT samples. The Enrichr tool was used for this analysis, and identified 21 pathways as significantly cold-responsive in fetal BAT samples (Supplementary Tables 3 and 4). Top 6 significantly regulated pathways in fetal BAT samples are listed in Fig. 4A, along with their significance levels (negative logarithm of the p-value). Several pathways related to muscle function such as *myofibril assembly*, *muscle contraction*, and *skeletal muscle cell differentiation* as well as metabolic pathways such as *fatty acyl-CoA biosynthetic process* were among the significant pathways. MA plots comparing pathway gene expression (blue) to non-pathway genes (light blue) were constructed for *muscle contraction*, *MyoD1 target,* and *fatty acyl-CoA biosynthetic process* pathways, as shown in Fig. 4B. Notably, the majority of genes contributing to *muscle contraction* and gene-targets of *MyoD1* transcription factor was downregulated in maternal cold-exposed fetal BAT samples. Decreased expression of 8 out of the 14 genes in fetal BAT samples was further confirmed by quantitative qPCR analysis (Fig. 4C). MyoD1 has been shown to be a key molecular inhibitor of brown adipocyte development [22]. Expression of MyoD in brown preadipocytes suppresses adipogenesis, whereas loss of MyoD in myoblasts promotes brown adipogenic differentiation [22]. Thus, downregulation of MyoD-target genes in 18 °C fetal BAT likely suggest that maternal cold exposure enhances brown adipogenesis in fetal BAT by suppressing the myogenic lineage in bidirectional progenitors that give rise to both brown adipocytes and myoblasts. In line with this, gene members of *fatty acid metabolism* pathway were selectively upregulated in 18 °C fetal BAT compared to 28 °C fetal BAT (Fig. 4B), suggestive of enhanced lipid storage in fetal BAT during maternal cold exposure. Increased expression of 4 out of the 7 genes in fetal BAT samples was further confirmed by quantitative qPCR analysis (Fig. 4D). However, we did not detect a significant increase in Ucp1 gene expression by maternal cold exposure (Fig. 4D). This may be due to no demand for fetal BAT thermogenesis in thermally stable intrauterine environment. Ucp1 gene expression is highly responsive to cold and markedly upregulated within several minutes of cold exposure [23]. Expression of adipogenic genes, Pparg and Fabp4, was not different in fetal BAT between the groups (Fig. 4D).

### Differential gene expression analysis of the placenta

**Fig. 5:**
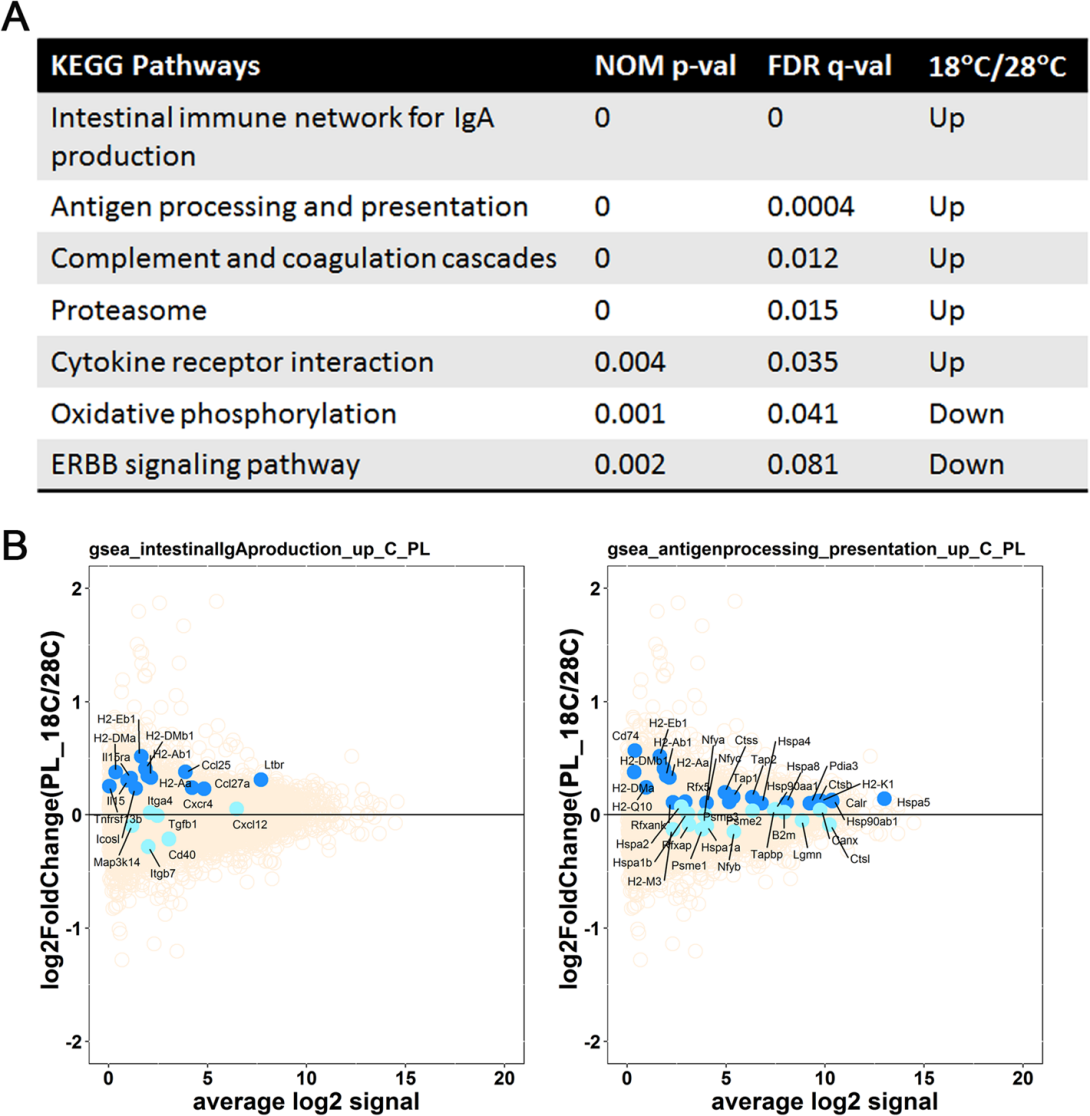
Effect of maternal cold exposure on placental gene expression. (**A**) Gene set enrichment analysis (GSEA) showing the pathways enriched in upregulated and downregulated genes in the placenta in a cold-dependent manner. (**B**) Mean-average (MA) plot analysis. Non-pathway genes are shown in tan, pathway genes contributing to pathway enrichment are shown in blue, and pathway genes not contributing to pathway enrichment are shown in cyan

An analogous pathway enrichment analysis via GSEA of placental samples revealed that 8 pathways were upregulated and 3 pathways were downregulated in 18 °C placental samples compared to 28 °C placental samples (Supplementary Table 2). Top 5 upregulated and top 2 downregulated pathways are listed in Fig. 5A. These encompass a variety of functions including *antigen processing and presentation*, *complement and coagulation cascades*, *proteasome, oxidative phosphorylation*, *ERBB signaling pathway*, etc. A comparison of the significant pathways in fetal BAT and placenta identified some interesting similarities and differences. The *proteasome* pathway was upregulated upon maternal cold exposure in both fetal BAT and placenta samples. The *ERBB signaling* pathway was downregulated by maternal cold exposure in both fetal BAT and placenta samples. However, both the *complement and coagulation cascades* and *oxidative phosphorylation* pathways were oppositely regulated in the fetal BAT and placental samples. While the *complement and coagulation cascades* pathway was downregulated in 18 °C fetal BAT and upregulated in 18 °C placenta, the *oxidative phosphorylation* pathway was upregulated in 18 °C fetal BAT but downregulated in the placenta under similar conditions. The MA plots in Fig. 5B show the distribution of pathway vs. non-pathway gene expression for two pathways related to immune function that are activated by maternal cold exposure.

**Fig. 6:**
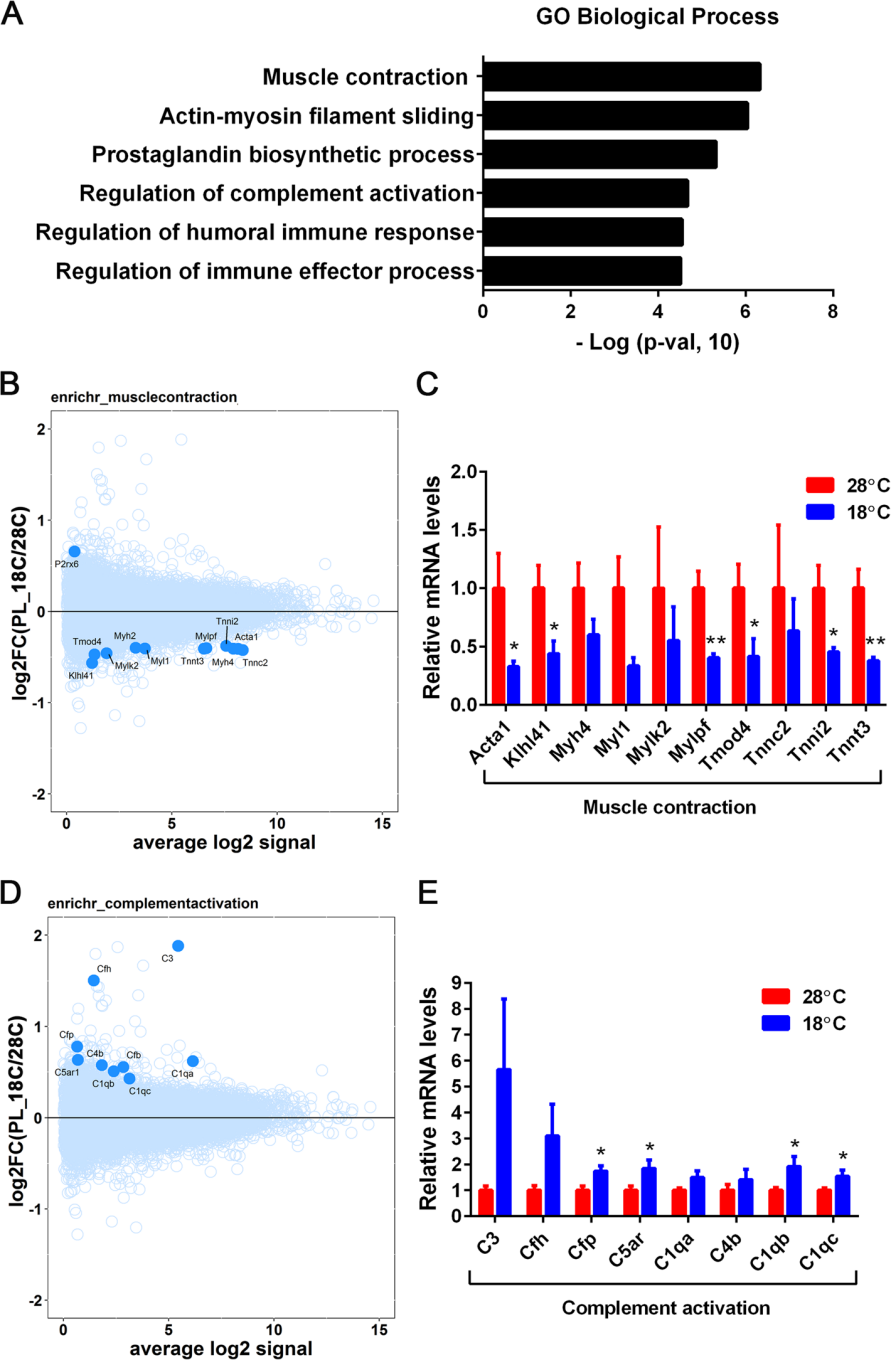
Pathways enriched in differentially regulated genes in maternally cold-exposed placenta. (**A**) Gene ontology (GO) analysis. 302 differentially regulated placental genes with a nominal p-value ≤0.05 (18 °C vs 28 °C) were used as input for Enrichr. (**B**, **D**) MA plot analysis. Pathway genes are shown in blue and non-pathway genes shown in cyan. (**C**, **E**) Quantitative real-time PCR analysis of 28 °C- and 18 °C-placental samples (n = 6 per group). Data are presented as the mean ± SEM. *P < 0.05 determined by Student’s *t* test

Enrichr based pathway over-representation analysis of 302 differentially expressed placental genes (nominal *p*-value ≤0.05) identified 37 pathways as significant at FDR < 0.1 (Supplementary Tables 3 and 4). Top 6 significantly regulated pathways in placental samples are shown in Fig. 6A and represent diverse functions such as *muscle contraction*, *actin-myosin filament sliding, prostaglandin biosynthesis*, *regulation of complement activation,* and *regulation of immune effector processes*. MA plots were generated for *muscle contraction* and *regulation of complement activation* pathways, as shown in Fig. 6B and D. Genes contributing to enrichment of the *muscle contraction* pathway were predominantly downregulated in maternal cold-exposed placental samples (Fig. 6B). Decreased expression of 6 out of the 10 genes was further confirmed by quantitative qPCR analysis (Fig. 6C). In contrast, gene-members of the *complement activation* pathway appear to be upregulated in maternal cold-exposed placental samples (Fig. 6D), similar to the upregulation of the *complement and coagulation cascades* pathway in GSEA (Fig. 5A). Increased expression of 4 out of the 8 genes involved in the *complement activation* pathway was further confirmed by quantitative qPCR analysis (Fig. 6E).

## Discussion

Female mice exposed to 18 °C during gestation activated BAT thermogenesis as evidenced by a marked increase in Ucp1 gene expression in maternal BAT. Our transcriptome analyses of placenta and fetal BAT further showed that environmental cold temperature sensed by the mother signals to the fetus and influences fetal brown adipogenesis. Maternal cold exposure was associated with substantial downregulation of myogenic genes and upregulation of genes involved in de novo lipogenesis and lipid metabolism in fetal BAT. A recent study by Son et al. highlighted that maternal exercise releases a muscle-secreted exerkine apelin, which in turn enhances fetal brown adipogenesis by inducing DNA hypomethylation in the Prdm16 promoter [24]. Given that cold-activated BAT secretes a number of batokines that regulate BAT itself or act on other organs by paracrine and endocrine mechanisms [25, 26], it would be interesting to determine if maternal batokine(s) secreted from cold-activated BAT to fetal circulation plays a role in brown adipogenesis in fetal BAT. Fatty acids mobilized from lipid droplets are a primary energy source for UCP1-mediated thermogenesis in BAT [2, 27]. Cold-dependent upregulation of lipogenic gene expression in fetal BAT may thus be a strategy for storing fat that can be immediately used by neonates for UCP1-mediated thermogenesis after birth. Further studies will be required to determine if cold-dependent changes in fetal BAT gene expression influence BAT thermogenic capacity of offspring.

Cold exposure of pregnant mice during gestation induced increased expression of genes involved in complement activation in the placenta. Exposure of pregnant rats to cold (4 °C) during late pregnancy has been shown to increase plasma corticosterone levels [28] and induce inflammation and apoptosis in the placenta [29]. The complement system, which is an integral part of innate immunity [30], is important for normal placentation [31] and plays a role in facilitating phagocytosis for clearance of placenta-derived particles and apoptotic cells [32]. Further investigation will be required to determine if increased expression of genes involved in the complement system is an adaptive response to protect the placenta against cold stress-induced inflammatory damage.

Maternal cold exposure also induced downregulation of genes involved in muscle contraction and actin-myosin filament sliding in the placenta. Vascular smooth muscle cells in the medial layer of the vessel wall play a pivotal role in regulating the contraction and dilation of the vasculature [33]. Human placental villi have also been shown to possess extravascular myofibroblasts and smooth muscle cells that express non-muscle-type isoforms of contractile proteins [34–36]. Longitudinal contraction and relaxation of both the vascular smooth muscle cells and extravascular myofibroblasts and smooth muscle cells running longitudinally along the fetal vessels of the placental villi are suggested to regulate the intervillous volume, maternal perfusion, and fetal villous blood flow [34]. Murine uteroplacental and fetoplacental arteries have contraction and relaxation properties, responding to vasoactive agents [37], although the presence of extravascular myofibroblasts and smooth muscle cells in the murine placenta remains to be determined. Further studies will be required to investigate if cold-dependent downregulation of genes involved in muscle contraction and actin-myosin filament sliding in the placenta influences fetal villous blood flow.

Compelling evidence from both animal models and humans has demonstrated sex-dependent structural, functional and transcriptional differences in the placenta [38]. Fetal sex was not determined in the current study. Given the presence of sexual dimorphism in BAT mass and function in adult rodents and humans [39], fetal sex will need to be considered in future studies into fetal BAT development.

## Conclusions

In summary, our findings using an unbiased high-throughput RNA-seq approach provide evidence that maternal cold exposure can modulate the transcriptome of placental and fetal BAT tissues. The ramifications of the observed gene expression changes warrant future investigation.

## Methods

### Animal studies

C57BL/6 mice were purchased from the Jackson Laboratory (Bar Harbor, ME), housed in standard conditions (22–23 °C; 12-h light/12-h dark cycle), and maintained on a regular chow diet (5001, LabDiet, St. Louis, MO) with ad libitum feeding. For pregnancy experiments, fourteen female mice at 9 weeks of age were randomly divided into two groups and acclimated at near thermoneutrality (28 °C; *n* = 7) or mild cold temperature (18 °C; n = 7) for 1 week prior to mating with males. Mice were monitored by visual inspection of their behavior twice a day to ensure acclimation at mild cold temperature (18 °C). After mating with males, pregnancy was determined by copulatory plug occurrence and assigned the embryonic day 0.5. Pregnant mice were maintained at their respective temperature, monitored for their body weight during pregnancy, and euthanized at E18.5 by carbon dioxide asphyxiation followed by cervical dislocation. After excising the gravid uterus, individual fetoplacental units were dissected free from maternal decidua. Fetuses were immediately euthanized by decapitation, and fetal BAT was collected from the dorsal neck area. These euthanasia methods are in accordance with the established recommendations of the American Veterinary Medical Association (AVMA) Guidelines for the Euthanasia of Animals. All animal care and experimental procedures performed were approved by the Institutional Animal Care and Use Committee of the Pennington Biomedical Research Center. All animal experiment reporting adhered to the ARRIVE guidelines [40]. Measurement of placental and fetal weight was performed blinded to the experimental condition to minimize bias. The number of animals for each group in this study was estimated using a two-sided t-test (G*Power v3.1.9.2) [41] with a power set at 80% and a significance level set at 0.05 for detecting a difference in gene expression between two groups.

### RNA isolation and library preparation

Total RNA from placenta (6 biological replicates per temperature) and fetal BAT (6 biological replicates per temperature generated by pooling BAT from 3 fetuses due to their very small size) samples was isolated using Tri-Reagent (Molecular Research Center) followed by purification on an RNeasy column (Qiagen) with DNase treatment. The quantity and quality of purified RNA were determined using an Agilent 2100 Bioanalyzer, and only samples with a RIN number (RNA integrity number) greater than 7.0 were processed further. The QuantSeq libraries were prepared using Lexogen’s QuantSeq 3′ mRNA-Seq Library Prep Kit for Illumina (Lexogen), according to the QuantSeq User Guide protocol. To reduce bias in sample analysis, RNA isolation and library preparation were performed blinded to the experimental condition. The pooled libraries were then sequenced using the Illumina NextSeq 500 (Illumina). Primary analysis of sequencing reads was performed using the Lexogen Quantseq pipeline V1.8.8 on the Bluebee platform for quality control, mapping, and raw read count tables (https://lexogen.bluebee.com/quantseq/). Sequence reads were aligned to the GRCm38 mouse reference genome (https://www.ncbi.nlm.nih.gov/assembly/GCF_000001635.20/). Library preparation and sequencing were done at the Pennington Biomedical Research Center’s Genomics Core.

### Outlier sample detection

Potential sample outliers were identified via principal components analysis (PCA) using the *prcomp* function in base R. Raw counts obtained from RNA sequencing were variance stabilized and log2 transformed prior to PCA analysis. A scatterplot of the first two principal components (PCs) was generated to visually inspect for outliers. Additionally, a statistical test for outlier detection was performed by calculating whether, for each of the 24 principal components, the absolute PC score of a sample exceeded the median score for all samples by 5 times the median absolute deviation. Based on these criteria, sample RT28BAT.1 was deemed an outlier for PC2, C18BAT.3 was an outlier for PC3, and sample RT28PL.6 was an outlier for PC8. However, since the first 2 PCs captured most of the observed variation in gene expression, we only removed the PC2 outlier sample, RT28BAT.1 from further analysis.

### Transcriptome analysis

Differential gene expression analysis on the RNA sequencing reads was performed via the Empirical Analysis of Digital Gene Expression, or edgeR package in R. Low expressing genes with a sum of < 5 counts per million (cpm) across all samples were filtered out. Raw counts for the remaining genes were adjusted for sequencing library size via the trimmed mean of M-values (TMM) method available in edgeR. Inter-library variation in gene expression (dispersion) was estimated from the normalized gene expression counts based on the negative binomial distribution and empirical Bayes methods. Differential gene expression was identified by fitting a quasi-likelihood negative binomial generalized log-linear model to count data [42]. Analysis of pathway enrichment between 28 °C- and 18 °C-exposed groups was conducted via GSEA-preranked method with no prior gene filtering [43] or via Enrichr [44] by using a pre-filtered list of differentially expressed genes (nominal *p*-value ≤0.01 and absolute fold-change ≥1.5 for fetal BAT samples; nominal *p*-value ≤0.05 for placenta samples). A modified pathway database, containing KEGG as well as user-defined custom pathways, was used for GSEA-preranked analysis. For Enrichr, pathways from KEGG, Wikipathways and Gene Ontology, as well as putative transcription factor target genes from Chipseq studies were queried. Pathways with FDR ≤0.1 were considered significant for GSEA analysis, whereas for Enrichr the pathway significance level was set at an adjusted p-value ≤0.05. Expression of genes within a subset of the significant pathways was visualized via mean-average (MA) plots. Gene expression data (GSM4593259–4593282) have been deposited in the Gene Expression Omnibus (GEO) database with accession number GSE151905 (https://www.ncbi.nlm.nih.gov/geo/query/acc.cgi?acc=GSE151905).

### Validation of RNA-Seq results by quantitative real-time PCR

To validate differential expression of genes that are enriched in the biological pathways identified by pathway over-representation analysis of 230 differentially expressed fetal BAT genes (nominal p-value ≤0.05 and absolute fold-change ≥1.5) and 302 differentially expressed placental genes (nominal p-value ≤0.05), we selected top 2 pathways per tissue based on their high significance levels. 1 μg of RNA samples was reverse transcribed using oligo dT primers and M-MLV reverse transcriptase (Promega), and 4 ng of cDNA were amplified by specific primers in 10ul reaction using SYBR Green Supermix (Bio-Rad) on an Applied Biosystems 7900 (Applied Biosystems). For each gene, relative abundance of mRNA was determined after normalization to cyclophilin by the 2^-ΔΔCt^ method.

### Statistical analysis

All bar graphs were created by using the Prism 6 software (GraphPad Software, San Diego, CA, USA) and student *t* test was used to compare the differences between groups. Data are presented as mean ± SEM. Values of *P* < 0.05 were considered statistically significant.

## Supplementary Information

**Additional file 1.**

**Additional file 2.**

**Additional file 3.**

**Additional file 4.**

## Data Availability

The datasets generated and analyzed during the current study (GSM4593259–4593282) have been deposited in the Gene Expression Omnibus (GEO) database with accession number GSE151905 (https://www.ncbi.nlm.nih.gov/geo/query/acc.cgi?acc=GSE151905).

## Abbreviations

ACACA: Acetyl-CoA carboxylase alpha
ACC1: Acetyl-CoA carboxylase 1
ACC2: Acetyl-CoA carboxylase 1
ACSL5: Acyl-CoA synthetase long chain family member 5
ACTA1: Actin alpha 1, skeletal muscle
ACTC1: Actin alpha cardiac muscle 1
ADRB3: Adrenoceptor beta 3
ANKRD1: Ankyrin repeat domain 1
C1QA: Complement C1q A chain
C1QB: Complement C1q B chain
C1QC: Complement C1q C chain
C3: Complement C3
C5AR: Complement C5a receptor
CACNG1: Calcium voltage-gated channel auxiliary subunit gamma 1
CASQ2: Calsequestrin 2
CCDCL141: Coiled-coil domain containing 141
CFH: Complement factor H
CFP: Complement factor properdin
CHRNA1: Cholinergic receptor nicotinic alpha 1 subunit
CHRND: Cholinergic receptor nicotinic delta subunit
CPT1B: Carnitine palmitoyltransferase 1B
CPT2: Carnitine palmitoyltransferase 2
DGAT1: Diacylglycerol O-acyltransferase 1
DIO2: Iodothyronine deiodinase 2
ELOVL3: Elongation of very long chain fatty acids protein 3
ELOVL6: Elongation of very long chain fatty acids protein 6
F2: Coagulation factor 2, thrombin
FASN: Fatty acid synthase
GK: Glycerol kinase
KLHL41: Kelch like family member 41
LCAD: Long-chain acyl-CoA dehydrogenase
MCAD: Medium-chain acyl-CoA dehydrogenase
MYH4: Myosin heavy chain 4
MYH8: Myosin heavy chain 8
MYL1: Myosin light chain 1
MYLK2: Myosin light chain kinase 2
MYLPF: Myosin light chain, phosphorylatable, fast skeletal muscle
MYOM2: Myomesin 2
PGC-1α: Peroxisome proliferator-activated receptor gamma coactivator 1-alpha
RAPSN: Receptor associated protein of the synapse
SCAD: Short-chain acyl-CoA dehydrogenase
SCD1: Stearoyl-Coenzyme A desaturase 1
SCD2: Stearoyl-Coenzyme A desaturase 2
SGCA: Sarcoglycan alpha
SMIM5: Small integral membrane protein 5
TMOD4: Tropomodulin 4
TNNC2: Troponin C2, fast skeletal type
TNNI2: Troponin I2, fast skeletal type
TNNT3: Troponin T3, fast skeletal type
TRMT44: tRNA methyltransferase 44 homolog
UCP1: Uncoupling protein 1
